# The economic impact of open science: a scoping review

**DOI:** 10.1098/rsos.250754

**Published:** 2025-09-17

**Authors:** Lena Tsipouri, Sofia Liarti, Silvia Vignetti, Izabella Martins Grapengiesser

**Affiliations:** ^1^ National and Kapodistrian University of Athens, Athens, Greece; ^2^ OPIX PC, Maroussi, Greece; ^3^ CSIL, Milan, Italy; ^4^ Technopolis Consulting Group, Brussels, Belgium

**Keywords:** open science, open access, open data, open source software, open methods, economic impact

## Abstract

This article summarizes a comprehensive scoping review of the economic impact of open science (OS), examining empirical evidence from 2000 to 2023. It focuses on open access (OA), open/FAIR data (OFD), open source software (OSS) and open methods, assessing their contributions to efficiency gains in research production, innovation enhancement and economic growth. Evidence, although limited, indicates that OS accelerates research processes, reduces the related costs, fosters innovation by improving access to data and resources and this ultimately generates economic growth. Specific sectors, such as life sciences, are researched more and the literature exhibits substantial gains, mainly thanks to OFD and OA. OSS supports productivity, while the very limited studies on open methods indicate benefits in terms of productivity gains and innovation enhancement. However, gaps persist in the literature, particularly in fields like citizen science and open evaluation, for which no empirical findings on economic impact could be detected. Despite limitations, empirical evidence on specific cases highlight economic benefits. This review underscores the need for further metrics and studies across diverse sectors and regions to fully capture OS's economic potential.

## 1. Introduction

The aim of this article is to review and summarize the literature that provides evidence for the economic impact of open science (OS) and identifying gaps where further research is needed. This review is a complement to others on academic [1] and societal [2] impacts of OS.

OS has a long history, and its definitions have evolved. In this paper, we use the OECD definition: ‘*Open science refers to efforts by researchers, governments, research funding agencies or the scientific community itself to make the primary outputs of publicly funded research results – publications and the research data – publicly accessible in digital format with no or minimal restriction as a means for accelerating research* ’ [3]. This openness can be applied across the full research workflow, bringing greater transparency, accessibility and participation through practices like open protocols and methods, co-creative and participatory approaches like citizen science, sharing of open data and code, transparent evaluation via open peer review and making scholarly publications open access (OA). The academic community pioneered OS and a large number of declarations^1^ and funders’ policies^2^ have been adopted. These initiatives aimed to make research freely accessible to anyone with internet access, driving advances in all scientific fields.

Benefits of OS go beyond the scientific community [4]. Since endogenous growth theory considers knowledge an essential driver of innovation, productivity enhancement and long-run economic expansion [5] reducing the barriers to its access and stimulating wider dissemination was seen as essential to accelerate and facilitate this positive process. UNESCO describes OS as a ‘critical accelerator for the achievement of the United Nations Sustainable Development Goals and a true game changer in bridging the science, technology and innovation gaps and fulfilling the human right to science’ [4].

The economic rationale for OS builds on the idea of knowledge as a public good^3^ [6,7]. This is related to making its access and dissemination open. The economic justification for making publicly funded research results openly available stems largely from Paul David [8–10]. Suber [11] further contended that taxpayers should not be charged twice for access to publicly funded research. This line of reasoning led to growing interest in assessing the economic impact of open versus closed science, prompting a wave of research on the benefits and challenges of mandating open availability of publicly funded scientific results.

Since then, a substantial body of theoretical studies and models has emerged, examining the benefits of OS through the lens of economics, aiming to demonstrate the benefits of opening up research results. Academic research papers on the economics of technological change make strong theoretical arguments in support of OS [12–14]. Overall, the main economic rationale is an increase in efficiency in science production [3]. By reducing the cost of reproducing, collecting and processing data, OS is then thought to foster research productivity [1].

Some authors theorize the economic value of OS using applications and examples from their respective industries, like McManamay & Utz [15] for fisheries, Harding [16] for medicine and Chan [17] on the economics of the transition to the OS Model. However, such papers provide no precise quantitative evidence in support of the argument that free-of-cost access to new knowledge accelerates technological progress. Arguments for the economic benefits of OS include the willingness of individual firms and government services to benefit from research results or cooperate with academia or other companies [18]. In addition, expressing views or forming alliances for FAIR (findable, accessible, interoperable and reusable) data [19] are considered as evidence of the economic value companies—innovative enterprises in particular, expect from OS.

In addition to these positive arguments for the economic impacts of OS, others have flagged potential economic downsides. As an example, Mueller-Langer & Andreoli-Versbach [20] used a theoretical model to find that while mandatory disclosure of research data is an essential feature for credible empirical work, it may come at a cost. They claimed that authors might invest less in data generation if they are not the full residual claimants of their data after the first journal publication and they might ‘strategically delay’ the time of submission of papers to fully exploit their data in subsequent research. This means that the welfare effects of universal mandatory data disclosure are ambiguous and risk being welfare-reducing, unless accompanied by appropriate incentives which deter strategic delays. No empirical evidence is however provided to support this argument.

Given this wealth of theoretical claims, assessment of the evidence supporting or refuting them is essential for orienting and finetuning future OS policy. Previous reviews have indicated that this evidence base is thin. Other reviews have considered the impact of OS as a whole (i.e. academic, societal and economic impacts [21]) or economic impact of specific components, such as OA [22], open/FAIR data (OFD) [23] and citizen science [24]. One previous study, Fell’s [25] ‘rapid review’ targeted the economic impact of OS specifically. While it found some evidence that OS enabled reductions in costs (access, labour and transaction) or could enable new research, products and service, it also admits that evidence is only indicative, being ‘patchy and diverse’. In this literature a prevalent theme is that, despite abundant theoretical arguments, the potential economic impacts of OS are still poorly documented. More evidence on how OS partnerships drive innovation and economic growth is urgently needed [26] to sustain growing support for OS and to strengthen the case against critics.

Creating such evidence is the aim of the PathOS project (https://pathos-project.eu), an EC-funded initiative to better understand and measure OS impacts and their causal mechanisms. Initiation of this work included a series of scoping reviews of current available evidence. We hereby present our scoping review of the economic impact of OS, complementing already published reviews on academic [1] and societal [2] impacts. This review was conducted to both update the available evidence and align the synthesis with an impact pathway logic. By structuring the findings around main OS dimensions and causal relationships between different impact aspects, this review provides a systematic and structured approach to understanding the economic implications of OS. This perspective enhances clarity on how various dimensions of OS contribute to economic outcomes, advancing and complementing existing literature.

Our main research question: *What evidence exists in the literature regarding the effect of OS on the economic impact of research?* This can be decomposed into secondary research questions:

— SRQ1: What types of positive or negative, direct or indirect impacts are observed?
— SRQ2: What kinds of mechanisms produce them?
— SRQ3: What specific enabling and/or inhibiting factors (drivers and barriers) are associated with these impacts?
— SRQ4: What knowledge gaps emerge from this analysis?

## 2. Methods

The study followed a systematic approach, based on PRISMA-ScR, to answer its research questions through four key stages: identifying relevant studies, selecting those that met eligibility criteria, extracting data into a data chart, and then summarizing and reporting the findings. The study’s protocol [27] was pre-registered on 31 October 2022, and updated with an addendum [28] on 29 June 2023, which provided additional details regarding the search process for grey literature and snowballing techniques. Both documents are available on the Open Science Framework (OSF) platform (https://osf.io). Any deviations from the original plan are outlined in electronic supplementary material, S1.

### 2.1. Identifying relevant studies

The Scopus and Web of Science (WoS) databases were used to identify relevant peer-reviewed literature published in English from 1 January 2000, up until 8 November 2022, when the results were collected. The search included broad terms related to impact (impact, effect and outcome), and key terms specifically focused on economic impact, including economic impact, financial/monetary impact, cost-benefit analysis (CBA), benefit-cost analysis (BCA), input-output, return on investment, patenting, innovation, new products, efficiency gains and savings^4^ (see table 1).

**Table 1.** Primary search strings for Web of Science (WoS) and Scopus.

Web of Science	(TI= (‘open scien*’ OR ‘science 2.0’ OR ‘open data’ OR ‘FAIR data’ OR ‘open access’ OR (‘open code’ OR ‘open software’ OR ‘open tool*’) OR ‘open method*’ OR ‘citizen science’ OR ‘open peer review’ OR ‘open metric*’) OR AB= (‘open scien*’ OR ‘science 2.0’ OR ‘open data’ OR ‘FAIR data’ OR (‘open code’ OR ‘open software’ OR ‘open tool*’) OR ‘open method*’ OR ‘citizen science’ OR ‘open peer review’ OR ‘open metric*’ OR ‘open access publ*’ OR ‘open access paper*’ OR ‘open access journal*’ OR ‘open access book*’)) AND TS = ((impact* OR effect* OR outcome*) AND (econom* OR financ* OR cost* OR mone* OR cba OR bca OR ‘input-output’ OR ‘return on investment’ OR ‘patent*’ OR ‘innovation*’ OR ‘product*’ OR ‘efficiency gain*’ OR ‘saving*’))
Scopus	TITLE-ABS (‘open scien*’ OR ‘science 2.0’ OR ‘open data’ OR ‘FAIR data’ OR (‘open access’ W/1 publ* OR paper* OR journal* OR book*) OR (‘open code’ OR ‘open software’ OR ‘open tool*‘) OR ‘open method*’ OR ‘citizen science’ OR ‘open peer review’ OR ‘open metric*‘) OR TITLE (‘open access’) AND TITLE-ABS-KEY ((impact* OR effect* OR outcome*) AND (econom* OR financ* OR cost* OR mone* OR cba OR bca OR ‘input-output’ OR ‘return on investment’ OR patent* OR innovation* OR product* OR ‘efficiency gain*’ OR saving*)) AND (PUBYEAR>1999) AND (LIMIT-TO (LANGUAGE, ‘English’))

During the second phase of the study, additional literature was identified through two approaches: a ‘snowball search’, which analysed citations from previously reviewed or included studies, and a systematic search of ‘grey literature’ which included non-peer-reviewed materials from organization websites (e.g. EC, UNESCO, OECD, UKRI, etc.). This phase also involved searching preprint servers (e.g. OSF) and conducting general web search using platforms like Google. Full documentation, including the code and data from these searches, is available in the shared dataset accompanying the study.

### 2.2. Selection of eligible studies

The selection process followed the PRISMA-ScR checklist, with specific inclusion criteria applied during both the title/abstract and full-text screening phases, as listed below:

— Studies must provide evidence of the economic impact of OS, which encompasses six OS types, namely OA, OFD, open methods, open code/software, citizen science and open evaluation.
— Studies must be conducted at either national or international levels.
— Studies must be published between 1 January 2000, and the search date.
— Studies must be written in English.
— Studies must have full-text availability.
— Studies must be either a research article, review article, conference paper or other peer-reviewed output, or a grey literature study from a recognized stakeholder.
— Can use any methodologies (quantitative, qualitative, mixed or others).

In addition, we specifically excluded the following:

— Concepts with partial similarities to open science, which some authors relate to OS. Although some argue that corporate participation in crowdfunding [29], open innovation [30] and academic-industry collaboration [31,32] signal support for OS, these are broader concepts. Investigating how OS interacts with collaborations is an intriguing topic for future research but lies outside the scope of this review.
— OFD from the public sector, other than research results, is excluded from our analysis, as this paper focuses solely on academic research results. A particular challenge was to decide whether to include economic evidence from repositories mixing open data regularly generated by the public sector, which partly include research results (e.g. Copernicus). The decision was based on the observation of the limited share of academic research results, which was our main scope, within the repository.
— Literature reviews: while not considered primary evidence, literature reviews played a critical role in guiding our snowballing technique, helping us identify relevant studies and prolific authors focused on the economic impacts of OS. When relevant, reviewed papers were included in our sample.
— Indirect economic impacts stemming from academic benefits: topics such as the costs of publishing and article processing charges are not explored.

The search in Web of Science (WoS) and Scopus yielded 7397 results, which were then screened as per the PRISMA-ScR methodology. Initially, obvious false positives were eliminated by reviewing the titles. The remaining records were merged, and duplicates were deleted, yielding 3538 entries. Following this, three researchers reviewed the titles and abstracts. Two researchers assessed each record, assigning a 'yes', 'no' or 'unsure' code to decide its inclusion. A third researcher assessed any data tagged as 'unsure' or with conflicting 'yes' and 'no' judgements and made the final decision on inclusion. During this process, the specific aspects of OS (e.g. OA, open methods, etc.) of each study were also recorded.

The full texts of the initially selected studies were collected using various methods, such as library access, inter-library loans and direct contact with authors. A total of 100 full texts were successfully retrieved and saved to a shared Zotero folder for reference management. Four researchers shared the studies and conducted a full-text screening, which led to the selection of one study for further analysis.

**Figure 1.**
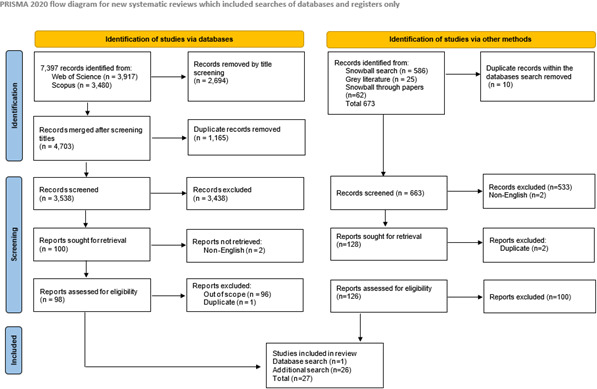
PRISMA-P flow diagram.

During the screening of snowball and grey literature, 128 studies were initially identified through title and abstract screening, which were narrowed down to 26, following full-text review. In total, across all search phases, 27 studies were included in the Scoping Review, as shown in the PRISMA-P chart (figure 1).

### 2.3. Extracting the data

The data extraction for the included studies was done collaboratively using an Excel file shared via Microsoft Teams. A pre-defined extraction form was used, covering study details, data sources, the study aims, types of impact, key findings, coverage and confidence assessments (see table 2). Each study was assigned to individual co-authors, responsible for extracting the relevant data.

**Table 2.** Categories extracted from included studies in the data charting process.

heading	description
author	name of author/s
date	date article sourced
title of study	title of the article or study
publication year	year that the article was published
publication type	journal, website, conference, etc.
doi/URL	unique identifier
exclusion	out of scope, non-English, duplicate
justification	if a study was deemed to be out of scope, justification had to be provided.
study details and design (if applicable)	type of study, empirical or review, etc. Notes on methods used in study (whether qualitative or quantitative, which population demographics studied, etc.)
types of data sources included	detail the data sources
study aims	overview of the main objectives of the study
relevance to which aspect of open science	open access, open/FAIR data, open methods, open code/software, citizen science, open evaluation
relevance to which aspect(s) of economic impact	efficiency gains, enhancing innovation, economic growth
key findings	noteworthy results of the study that contribute to the scoping review question(s)
coverage	optional field to note any relevant information about the level of coverage of the study, e.g. only specific countries, disciplines, demographics covered
confidence assessment	optional field to note any concerns about reliability/generalizability of findings (e.g. conflict of interest, potential biases, small sample sizes or other methodological issues) within the study

Economic impact categories were identified in an iterative way from the initial extraction through data screening and further analysis to better align with literature findings. This process led to the identification of three key categories: efficiency gains, enhancing innovation and economic growth.

### 2.4. Limitations

Several limitations should be acknowledged. First, the review adhered to the PRISMA protocol, which comprises well-defined inclusion criteria. While this enhances the robustness of the approach, it may limit the inclusiveness of the exercise. For example, only studies published in English were considered, potentially excluding relevant research in other languages. In addition, grey literature was initially excluded and added only in a second phase based on a snowball strategy. Second, the scope of the review was limited to examining the economic impact of OS on users, such as researchers, companies and policymakers. Consequently, the effects of OS on the publishing sector were not within the review’s focus. While the publishing industry is an important stakeholder in the OS ecosystem, the decision to exclude its economic dynamics was made to ensure a clear and targeted analysis of user-centred impacts.

As a result, this scoping review did not aim to critically and systematically assess the robustness of the evidence or the methodological rigour of the included studies. As such, the findings presented should be interpreted as a comprehensive overview of the available evidence rather than a definitive assessment of its reliability or causal validity. Despite these limitations, this review contributes to the understanding of the economic implications of OS by providing a structured synthesis of existing research.

### 2.5. Summarizing and reporting the results

Data extraction results from the initial database search, grey literature and snowballed sources were compiled into a single Excel file, organized by OS aspects and shared on Microsoft Teams. Co-authors were assigned to summarize and narratively report these results in a shared Microsoft Teams document, organized by OS and economic impact aspects, and then refine it into the final version.

## 3. Results

### 3.1. Overview

**Figure 2.**
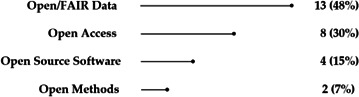
Number of studies reporting economic impact of open science by relevance to aspects of open science.

Our analysis draws on evidence from 27 studies, as illustrated in figure 2. The economic impacts identified from the literature are classified according to two dimensions:

— The *open science dimension*. Empirical evidence was found for:
  — *Open/FAIR data* (13 papers, 48% of OS type instances)**.**
  — *Open access* (eight papers, 30% of OS type instances).
  — *Open source software* (four papers, 15% of OS type instances).
  — *Open methods* (two papers, 7% of OS type instances).

while evidence is lacking for open evaluation and citizen science.

— The *economic impact dimensions,* explained as follows:
  — *Efficiency gains* occur because time and costs are saved through accessing and reusing knowledge, data, software or methods, rather than investing resources in recreating data or duplicating research.
  — *Enhancing innovation,* which as defined in the Oslo Manual, is ‘a new or improved product or process (or combination thereof) that differs significantly from the unit’s previous products or processes and that has been made available to potential users (product) or brought into use by the unit (process)’ [33]. In case OS reduces the cost of access and hence facilitates the use of knowledge for the creation of new product and processes creation, openness contributes to innovation.
  — *Economic growth* occurs when total factor productivity (or multifactor^5^) increases. It is measured by growth models based on the Cobb-Douglas production function [35–37].

### 3.2. Economic impact of open/FAIR data

Open data refers to research data that is freely accessible, usable and shareable by anyone. The FAIR principles ensure that data is well-structured, properly documented and can be effectively used by both humans and machines while respecting legal and ethical constraints. Our review identified 13 studies that assessed the economic impact of open data [13,38–49]. Among these, eight studies reported impacts on efficiency gains, four focused on enhancing innovation, and three highlighted impacts on economic growth. Two reported impacts on both efficiency gains and economic growth.

#### 3.2.1. Efficiency gains

Although speed is often emphasized as a critical factor, there is a scarcity of studies providing robust estimations of actual time savings and their impact. Yozwiak *et al.* [13] argued that sharing data accelerated the development of therapies, diagnostic tools and vaccines in both the public and private sectors during outbreaks, making the cases of Ebola epidemic in 2015 and H1N1 influenza in 2009. On contrary, a lack of or uncertainty in data-sharing practices during the Middle East respiratory syndrome (MERS) in 2012 slowed progress in addressing the challenges. However, the article did not provide quantitative evidence on the time saved and the related economic impact.

Available estimates of time savings rely on individual case studies. However, comparison is complicated by use of different metrics and a range of methods, even within individual cases. A number of studies were performed on UK open data services by Beagrie & Houghton [38–43] following the same methodology. For example, based on survey results and a mixed-method approach combining qualitative and quantitative analysis, Beagrie & Houghton [40] assessed the value and impact of the British Atmospheric Data Centre (BADC). The study found that the research efficiency reported by the users is high, but they estimated different values for it. A minimum value can be estimated by looking at the access costs defined as the time-related costs incurred by users to retrieve the data. Although these costs represent an expenditure, they can be interpreted as an implicit measure of the users’ willingness to pay. The underlying rationale is that if users are willing to incur these costs, it suggests that the efficiency benefits they will derive from accessing the data are at least equivalent to this value, thus establishing a lower bound. Access costs of BADC are estimated at £2.3 million per year. However, when users are asked to provide an explicit value, their responses often provide a different perspective, also depending on the survey design and method. Here a distinction must be drawn between ‘willingness to pay’ (WTP) to acquire something and ‘willingness to accept’ (WTA) compensation for forgoing it. The primary difference lies in that WTP is limited by an individual’s ability to pay, often determined by their disposable income, while WTA is not subject to such constraints. The choice between one approach or the other leads to different results. In the case of BADC, for example, the willingness to pay solicited through a contingent valuation (a survey-based economic technique for the valuation of non-market resources) shows a value of around £5.2 million. However, when asked about the willingness to accept, i.e. when capacity to pay is not considered, the value users place on BADC data and services rises to around £15 million per year. Finally, an upper bound of the efficiency gain was obtained by estimated time saving. Authors found that BADC data significantly improves research, teaching and studying efficiency, with estimated potential impacts ranging between £10 and 58 million per annum. Adjusted estimates also suggest efficiency savings closer to £10 million annually, still a substantial benefit of—double the operational and access costs. All these estimates provide distinct perspectives on the value of efficiency gains by, on the one side, enriching the overall understanding of their magnitude but, on the other, posing challenges to comparability and validity of results.

In a similar study on the Economic and Social Data Service (ESDS) of the UK Economic and Social Research Council (ESRC), Beagrie & Houghton [38] adopted the same approach, assessing economic impact at multiple levels. The aim of the ESDS is to encourage broader and informed use of data for social science research and education while ensuring the long-term preservation and accessibility of such data. Based on the result of two surveys, the authors found evidence on the use value calculated at £24 million per year. This value is quite close to the willingness to pay, calculated with a contingent valuation and resulting in a value of around £111 million per year. An upper bound of value to the users was instead estimated at around £111 million per year if considering the willingness to accept. The analysis adopted a counterfactual approach: approximately 70% of users surveyed thought that if ESDS had not existed they would not have been able to obtain the data in any other way; in addition, 58% reported that it was beyond their capacity to (re)create the data themselves. The extent to which these benefits are accurately reflected in the contingent valuation remains uncertain.

In 2013, Beagrie & Houghton [39] assessed and measured the value and impact of the Archaeology Data Service (ADS). As in previous studies, they used a mixed-method approach combining qualitative and quantitative analysis. The assessment of ADS data and services reveals direct use value to the ADS user community to £1.4 million per annum, while users' willingness to pay indicates a value of around £1.1 million per year and willingness to accept £7.4 million annually [39].

Finally, Beagrie & Houghton [42] showed that the services of the European Bioinformatics Institute of the European Molecular Biology Laboratory (EMBL-EBI) offering data openly are widely used and highly valued by their user community. The time users spent accessing data and services was valued at £270 million for 2015, while the willingness to pay was estimated at £322 million. The study was then updated in Beagrie & Houghton [43], employing multiple approaches in an attempt to look at more immediate/direct effects but also to wider impacts. It built on the results of a user survey which received almost 5000 responses. The use value is estimated at £5.5 billion per annum, a willingness to pay of £1.25 billion per annum and a willingness to accept equal to £68 600 per respondent, per annum. The study estimated the total efficiency savings from time spent using data from EMBL-EBI, as reported by users, to range between £2.6 billion and £11 billion per year.

The analysis of the global utilization of the Research Collaboratory for Structural Bioinformatics (RCSB) Protein Data Bank [44] echoed previous works done by Beagrie and Houghton and calculated the productive time researchers gain, associated with using RCSB-PDB data and services at $2.5 billion annually, measured by the time allowing researchers more freedom for other activities. The analysis built on website analytics (user numbers and levels of use) and on estimates made by Beagrie & Houghton [42].

Available evidence shows that efficiency gains associated with OFD do not come without costs. Data creation, curation and deposition are costly activities. Moreover, accessing data (e.g. searching, screening, downloading and interpreting data) also requires time. The screened studies consider these aspects and also provide estimates of costs. The interesting result is that, compared with the benefits, the net result is always positive, although with variation in terms of magnitude. Beagrie & Houghton [42] showed that stated benefits are around six times the annual operational cost of EMBL-EBI. By adding access costs borne by the users, the result remains positive. Estimated efficiency gains of ADS is about five times the combined operational, depositor and user access time costs [39]. Returns calculated on the BADC data and services equates a rate ranging from 4 to 12 times the costs [40].

#### 3.2.2. Enhancing innovation

Four studies investigated how OFD can enhance innovation. Using data on the Human Genome Project and the private firm Celera,^6^ a paper by Williams [45] estimated the impact of Celera’s gene-level IP on subsequent scientific research and product development. Genes initially sequenced by Celera were subject to intellectual property (IP) restrictions for up to two years but were made publicly available once re-sequenced by the public Human Genome Project. By mapping the timing of gene sequencing and Celera’s IP and linking them to scientific research and product development, the author found that the IP constraints reduced scientific research and related product development by approximately 20–30%. Though the analysis admittedly does not assess the welfare consequences, these findings indicate that Celera’s temporary IP protections had a lasting negative impact on innovation, compared with a scenario where Celera’s gene sequences had been in the public domain from the outset.

Using patents as a proxy for innovation, Arshad *et al.* [46] found, in a case study on the use of a specific molecule, that the open dissemination of the molecule significantly increased its adoption among a diverse and multidisciplinary research community. A comparative examination of patent landscapes for various drug candidates revealed that the public release of molecule data spurred greater innovation, as evidenced by a higher number of downstream patents associated with it. These findings challenge the assumption that open data drug discovery compromises commercial IP. Instead, it exemplifies how open data can drive economic benefits by reducing the costs of both drug discovery and commercialization.

Giovani [47] provided a detailed examination of how OFD influences innovation and IP strategies within the pharmaceutical industry. The study, based on a survey of 13 pharmaceutical firms, investigated the extent to which OFD (including both government and research results) affects companies' research activities or strategic monitoring, but also its impact of OFD on IP strategies. The findings revealed that pharmaceutical firms report leveraging OFD to complement proprietary data for research purposes, implement licensing-in and licensing-out strategies, map partnerships and networks among industry players, and identify key expertise for recruitment. Giovani argued that using and contributing to the OFD movement may change the approach of pharmaceutical firms to IP and innovation.

An alternative path to assess the innovation enhancing power of OFD is to apply a foresight methodology. Nielsen *et al.* [48], using workshops with industry and experts, found consensus among experts on the fact that open data accelerates innovation and enhances efficiency, potentially lowering product development costs and enabling better legislative and environmental decision-making. The panel of experts also agreed that open data can improve transport systems, enhance journey planning and reduce societal costs, but it demands strategic frameworks to balance its benefits with risks like job displacement and cybersecurity concerns.

#### 3.2.3. Economic growth

Three studies assessed the wider impacts generated by OFD on the economy as a whole, all of them relying on economic model simulations. A notable example is the study by Tripp & Grueber [49] which attempted to quantify the direct and indirect economic benefit of the abovementioned Human Genome Project providing open data on mapped sequences. The study concluded that between 1988 and 2010, the project and associated research and industry activity generated an economic impact of $796 billion, personal income exceeding $244 billion and 3.8 million job‐years of employment. Considering that the federal government invested $3.8 billion and that in 2010 alone the genomics‐enabled industry generated over $3.7 billion in federal taxes, the return on the investment was very high. The results related to the HGP are often cited as a benchmark for demonstrating the significant economic returns on investment in science. Some critics address the assumptions underlying the economic models which may lead to overestimations or lack sensitivity to alternative scenarios.^7^ There are also attribution problems about the extent to which the outcomes quantified can be directly linked to the HGP and not to parallel private and public investments in genomics and related fields.

Wider economic growth estimates were also attempted for smaller initiatives. Beagrie & Houghton [43], with a macro-economic model, estimated the return on investment in R&D made possible by EMBL-EBI data. The study distinguished between return on R&D facilitated or depending on EMBL-EBI managed data, the first equalling £2.2 billion annually and the second being £1.3 billion annually or £9 billion over 30 years in net present value. These figures demonstrate substantial increases from the previous 2015−2016 impact study (which estimated £920 million annually, or £6.9 billion over 30 years).

The analysis of the contribution to the local economy and the global utilization of the Protein Data Bank [44] estimated an impact of R&D expenditure of $1.1 billion annually, noting this as a conservative estimation. Over a period of 30 years, the estimated impact is valued at $8.0 billion.

In all these simulations, attribution problems remain when assessing impact far beyond the direct use of the data, since other confounding or facilitating factors may intervene along the causal chain of effects.

#### 3.2.4. Summary of the economic impact of open/FAIR data

The evidence collected is limited but convincing, since it is based on solid methodological design and empirical analysis. Evidence is particularly compelling in the case of life science research and applications to the pharma industry. Efficiency gains rise as researchers save both time and financial resources in conducting their research activity. Financial savings occur by avoiding expenses such as subscription fees to access the data necessary for their research. Time savings occur, not only because the search process is streamlined, but because researchers can also directly use existing data. Reproducing such data would require substantial time investment and the development and maintenance of adequate skills. Time savings can accelerate the uptake of research results by industries, which can in turn produce more innovation.

Using surveys and interviews, researchers can document and measure efficiency gains and innovation enhancement of individual initiatives. Most of the relevant included studies (see Appendix) come from large research centres or repositories and demonstrate a significant variance in the indicators calculated. In all cases efficiency gains are high, except for the Archaeology Data Service (ADS). However, no generalizations or comparisons can be made given the limited number of cases and their peculiarity. In addition, caution should be paid to assessing wider impacts because they are based on economic model simulations and may raise attribution problems.

### 3.3. Economic impact of open access

Open access is the provision of free access to peer-reviewed, scholarly and research information to all. Our review identified eight studies examining the economic impact of OA [51–58]. Of these, four studies highlighted efficiency gains, two emphasized innovation enhancement and two focused on economic growth impacts.

#### 3.3.1. Efficiency gains

Efficiency gains are achieved as researchers save both time and costs associated with accessing knowledge. OA enables them to explore a larger reservoir of information at no cost, allowing them to efficiently identify and utilize the resources most relevant to their research activities, saving time and money.

Houghton *et al.* [51] examined the levels of access to and utilization of research results by small and medium-sized enterprises (SMEs) in Denmark. Through an online survey and in-depth interviews with knowledge-based SMEs, the authors assessed the cost and value of specific innovations, the role research publications played in it, and to what extent barriers to access was influencing the innovation capacity of SMEs. They concluded that without access to academic publications, replicating the same outcomes would have taken an extra 2.2 years, leading to significant opportunity costs. This efficiency gain was not broken down between *open access* in our sense and *availability*; the latter being also considered as open access in a broader sense by industry, since the knowledge is not proprietary but can be accessed at a relatively small cost. However, their respondents extensively used OA journals and repositories (more than half of respondents used OA journal monthly or more regularly), hence the efficiency gains can be partly attributed to OA.

Acceleration in scientific production is measured by Coccia [52], with specific reference to COVID-19 research. According to the author, scientific output increased by about 1.2% each day in the period April 2020–June 2021. Among the key characteristics influencing this peculiar evolution of research production, the author lists OA to publications, which facilitated the rapid dissemination of findings and contributed to the acceleration of research production. This effect was claimed to be crucial in supporting discoveries in the research arena over a short period of time, such as the development of innovative drugs and new antiviral COVID-19 treatments. Comparisons with other research fields during the same period, such as lung cancer, show a velocity of 0.4 of daily growth of scientific production [52]. However, the author did not reach a monetary quantification of this gain and could not even identify to what extent the increased productivity attributed to the severity of the pandemic or to the degree of openness.

The economic benefits of OA are not confined to the business community. Look & Marsh [53] explored the impact of time savings associated with OA in the public sector, analysing both direct and indirect benefits. Based on case studies of selected organizations, they found that OA through both green and gold routes^8^ led to £28.6 million in direct cost savings for the UK public sector (£26 million in access fees and £2.6 million in time savings). Indirect benefits (such as improved decision-making and policy analysis) were suggested, but they were not quantifiable within the scope of this study.

Houghton *et al.* [54] conducted a comparative analysis of three models of scholarly publishing, concluding that the benefits of OA publishing are likely to outweigh its costs. Their study did not only investigate the academic impact for researchers, but assessed also the impact for funding agencies, research institutions, the publishing industry, research libraries and government agencies. While the transition to OA publishing involves initial costs and a delay before benefits are fully captured, the long-term gains are significant.

#### 3.3.2. Enhancing innovation

The impact of OA on innovation is challenging to quantify, because it is the accumulation of various sources of knowledge (open, closed, internal) that contribute to innovation over time. Hence the impact can only be assessed either by big data or by surveys, interviews and case studies. The former refers to *patent data analysis***,** which offers the advantage of extensive data coverage but is primarily relevant to industrial sectors with a high propensity for patenting. The latter provides a broader perspective but requires significant effort and is hard to translate into standardized indicators. Unfortunately, very few studies have been identified with robust quantitative evidence.

Bryan & Özcan [55] took advantage of the National Institutes of Health (NIH) mandating free online availability of funded biomedical research in 2008, which provided a unique opportunity to conduct counterfactual analysis on OA. A difference-in-differences estimation was performed to assess the propensity of patent citations for articles published before and after April 2008, with and without NIH funding. The authors employed a novel tool that measured extracted in-text citations rather than the commonly used front-page patent citations, which they believed to be more closely linked to the inventor’s knowledge. Using all US patent applications filed between 2005 and 2015, the model concluded that, after 2008, patents cited NIH-funded research 12–27% more frequently than before. The result was controlled for non-funded research, funded research in journals unaffected by the mandate, and academic citations. The findings suggest that the open-access mandate led to increased patentability, one of the most frequently used proxies for innovation in economic literature.

Using a more recent dataset of 22 million patent families from 2010 to 2020, Jahn *et al.* [56] matched publicly available data sources on patents and scholarly publications for a similar analysis. Unlike Bryan & Özcan [55], who focused on patents from a single country, they examined global patent data from Google Patents alongside scholarly data from Unpaywall, Europe PMC and arXiv. Their analysis showed that the share of OA among scientific non-patent references in patents was above the general trend of all non-patent references. Their findings provided more detailed insights into the impact of OA across countries and disciplines. By analysing non-patent literature citations, they found that patent families in the fields of Human Necessities, Chemistry & Metallurgy and Physics had an above-average open-access share compared with the overall average. By contrast, the fields of Textiles and Fixed Construction exhibited lower-than-average open-access shares. Geographical patterns also varied significantly. The US and the UK ranked highest in the percentage of open-access patent families, followed by technologically advanced OECD members, China, Russia and Taiwan.

#### 3.3.3. Economic growth

Houghton & Sheehan [57] developed a variation of the Solow–Swan growth model^9^ to calculate aggregate efficiency gains and social returns on research and development (R&D). In their model, income is a function of technology change, capital and labour, all contributing to growth. The authors applied three assumptions: (i) the use of an ‘accessibility’ parameter as the proportion of the R&D knowledge stock that is accessible to those who could use it productively, (ii) the use of an ‘efficiency’ parameter as the proportion of R&D spending that generates useful knowledge, (iii) that the move to OA leads to a one-off increase in both accessibility and efficiency. Based on the model specification and these assumptions, the authors calculated the potential returns on R&D investment for alternative scenario (separately for GERD and GovERD^10^), to a one-off increase in accessibility and efficiency in the OECD countries. They simulated the results using shares of returns to R&D for 25%, 40%, 50%, 60% and 75% and examined percentage changes in accessibility and efficiency for 1%, 2%, 5% and 10%. They considered that the choice of percentages depends on each country’s characteristics and hence should be left to national experts to assess which ones were most suitable for their country. The study demonstrated that, whether applied across the board or to sector specific research findings (e.g. OA to publicly funded research), there were substantial benefits to be expected from expanding OA. Using Germany with a GERD at $58.7 billion as a showcase and choosing for social returns to R&D of 50%, and a 5% increase in access and efficiency. Their model suggested that OA would generate an additional $3 billion. With the same shares of social returns to R&D and increase in access and efficiency Japan with GERD of $112.7 billion would yield a gain of $5.8 billion.

In a later study for Australia, using the same model, Houghton & Sheehan [58] introduced the difference of openness/closedness in the model’s variables. They introduced again the concepts of 'accessibility' and 'efficiency' into the traditional Solow–Swan model and estimated how OA could increase returns on investment in R&D. This time they broke down the impact of a one-off increase in accessibility and effectiveness between Government Expenditure on R&D, Higher Education Expenditure on R&D and the research funding by the Australian Research Council. Increased accessibility could lead to faster research processes, reduced duplicative research and a higher rate of commercialization of findings. Their estimates pointed to potential gains from openness: assuming 20% rate of return of R&D a 5% increase in accessibility and efficiency in Australia one could expect a yield of recurring annual gains of AUD 165 million (€101 million) from government R&D expenditures, AUD 111 million (€68 million) from higher education R&D, and AUD 12 million (€7 348 080 from Australian Research Council-funded research).

#### 3.3.4. Summary of the economic impact of open access

Evidence on economic impact of OA remains limited. We identified eight papers assessing it with different methods and models indicating evidence on efficiency gains through accelerating research and cost saving, as well innovation enhancement. OA seems to contribute to patenting knowledge more than non OA. At an aggregate level, a growth model helps calculate OA contribution to growth under different assumptions of efficiency growth thanks to increased accessibility.

### 3.4. Economic impact of open source software

Our review identified four studies assessing the economic impact of open source software (OSS) [60–63]. OSS is defined as ‘software that anyone can study, inspect, modify, and distribute freely under very limited restrictions such as attribution’ [64,65]. Platforms such as GitHub,^11^ GitLab and Bitbucket have emerged as essential infrastructures for facilitating collaboration. While numerous science-based OSS projects exist—Linux and Python being prominent examples—there is no clear means of distinguishing between software derived from academic research from OSS in general.

Science-driven software development prioritizes knowledge, research and openness, often at the expense of usability and long-term support. By contrast, commercially developed software emphasizes profitability, market demands and scalability, resulting in more polished but often less open solutions. There are no systematic statistics quantifying the distribution between science-based and commercially developed OSS. One of the rare estimates [63,66] suggests that academic institutions in the United States contribute nearly one-third of the total OSS output. However, the absence of strict criteria or taxonomies to differentiate between research-based and commercially developed software necessitated a highly selective approach in our choice of papers.

#### 3.4.1. Efficiency gains

Nagle [60] examined the firm-level productivity effects of non-pecuniary OSS, finding that its impact depends on whether firms possess complementary capabilities, such as skilled information technology (IT) personnel and supportive infrastructure. Firms with these capabilities experience a positive and statistically significant productivity impact. A 1% increase in the use of non-pecuniary OSS results in an increase in value-added productivity of between 0.002% and 0.008%. While these productivity increments may appear modest at the firm level, the aggregated economic impact across an industry or sector can be substantial, given the widespread adoption and reliance on OSS in IT-driven industries.

Conti *et al.* [61] analysed how startups, which utilize GitHub, can secure funding and reduce operational costs. Their study identified key mechanisms through which efficiency gains are realized, particularly in cost savings, as firms reduce expenditures on software development, licensing fees and time-to-market. To rigorously assess these impacts, the authors employed a difference-in-differences approach, leveraging firm-level data to compare outcomes before and after engagement with open source communities (OSCs).

Chesbrough [62] conducted a survey to examine the costs associated with OSS for large corporations, particularly Fortune 500 firms engaged in software development projects. The survey required participants to estimate costs and value comparisons for a specific ‘focal project', referring to a concrete software development initiative selected by the respondent. Participants were asked to calculate the cost of OSS access, which was defined as the sum of installation and maintenance expenses. For the focal projects identified, ‘almost two-thirds of [the respondents] reported that the benefits exceeded the costs, and only one-fifth reported that the perceived costs exceeded the benefits’ [62]. Additionally, 46% of respondents believed that developing the code internally would have cost at least twice as much as using OSS. Respondents also highlighted benefits related to faster development times.

#### 3.4.2. Enhancing innovation

Conti *et al.* [61] combined data from GitHub and Crunchbase to conduct a counterfactual analysis assessing the likelihood of funding for early-stage startups engaging with OSCs on GitHub compared with peers that were not similarly engaged. They analysed 160 065 US startups. Their findings indicated that engagement was associated with an increase of at least 36% in the likelihood of receiving funding, with the relationship being strongest for firms working on novel technologies. They concluded that by integrating OSS into their operations, firms can minimize operational costs while maximizing productivity and innovation opportunities.

The opportunity costs of engagement with OSS, as identified in their study, were primarily related to appropriability. Startups operating in less competitive markets benefit more from OSS, as they experience fewer pressures to protect intellectual property (IP), whereas firms in highly competitive industries perceive this as a significant challenge. OSS played a crucial role in technological development, particularly for startups working on novel technologies such as artificial intelligence (AI) and blockchain.

A report by the European Commission *et al.* [63] examined how OSS supports innovation processes in Europe. Their results, based on surveys and case studies, demonstrated that OSS fosters innovation through three key mechanisms: enabling collaboration, reducing barriers to entry and establishing *de facto* standards. These mechanisms are particularly impactful in sectors such as healthcare and information and communication technology (ICT), where the collaborative nature of OSS accelerates technological advancements and facilitates the development of socially relevant products. The authors estimated that a 10% increase in GitHub contributions by EU member states could lead to the creation of over 650 additional IT startups within the EU. By leveraging the collaborative and accessible nature of OSS, these startups further strengthen innovation ecosystems and contribute to economic dynamism.

#### 3.4.3. Economic growth

Only one report was identified [63]—which provided an estimate of the OSS-related impact on GDP. The authors found that within the EU, OSS contributed an estimated €65 to €95 billion to GDP in 2018. Using econometric modelling, they projected that a 10% increase in OSS contributions could boost GDP by an additional 0.4% to 0.6% annually. Unlike the respondents in Chesbrough’s survey, the dataset underlying these projections was predominantly composed of startups and micro-enterprises, which were strongly represented in the study.

The report concluded that OSS not only contributes to GDP growth but also enhances labour productivity within the EU. National contributions to OSS strengthen domestic competitiveness, as reflected in metrics such as exports and trade in value-added.

#### 3.4.4. Summary of the economic impact of open source software

Based on the synthesis of the limited number of papers examined for our review, OSS appears to have economic impacts at both microeconomic and macroeconomic levels.

At the microeconomic level, OSS contributes to efficiency gains by reducing costs and saving time. It also plays a crucial role in fostering innovation, particularly in start-ups and in technology-intensive sectors. At the macroeconomic level, the available evidence, though limited with one report for the EU market only, suggests that OSS enhances labour productivity by lowering software development costs, providing free and reusable tools and enabling a more efficient allocation of resources eventually leading to GDP growth.

However, unlike the cases of OFD, OA above and open methods discussed below, conclusions regarding the economic impact of OSS are constrained by the challenge of distinguishing the extent to which OSS is science-based.

### 3.5. Economic impact of open Methods

Open methods in OS refer to sharing the entire research process transparently. This means making research steps, data analysis and workflows publicly available so others can understand, reproduce and build upon the work. Researchers do not only offer free access to their publications and data but also reveal in detail how to replicate the methods they used for their research. Our review identified only two studies that assessed the economic impact of open methods [67,68], both of which suggested that open methods contribute to efficiency gains and innovation enhancement.

#### 3.5.1. Efficiency gains

Lee [67] addressed the problem of failure to rapid innovation in drug discovery, reporting on the establishment of the Structural Genomics Consortium (SGC), a UK-registered charity operating as a precompetitive public–private research partnership. SGC’s pharmaceutical partners provide funding and collaborate on scientific research, without retaining IP rights or exclusive access to findings. Lee’s findings in a case study of an SGC project, collaborating with GlaxoSmithKlein, showed that the adopted OA enabled significant timesaving by accelerating drug discovery. By using structure-guided methods, researchers rapidly developed highly potent and selective protein inhibitors and shared them with the scientific community without restrictions. He reported the design of a small molecule in 11 months, and half a year for compounds to be used by the community. The registration of the first clinical trial took place 16 months after the first seminal publication. This case suggests that OA accelerates scientific research and generates pioneering drug programmes and clinical studies faster.

#### 3.5.2. Enhancing innovation

Open methods do not only enhance innovation through efficiency gains as indicated by Lee’s [67] but also through sharing information on research methods. In a publication addressing innovation enhancement directly, Murray *et al.* [68] developed a theoretical model suggesting that openness lowers the cost of accessing the ideas of others and nurtures new and speculative research lines facilitating exploration that leads to innovation. They had the opportunity to test their model with a natural experiment on genetically engineered mice because two IP holders (out of four methods developed for four categories of genetically modified mice) agreed in 1998 and 1999 with NIH to significantly lower the cost of access to the method or to acquiring mice directly. Prior to the agreement developing new mice would require significant resources and cause delays of the order of 18 to 24 months. This impacted more than 50 mice that had been developed and disclosed in the scientific literature allowing the authors to estimate pre- and post-NIH agreement citation rates to the treated mouse-articles and the control group. Robustness tests were conducted and confirmed the absence of selection bias in the control group. Using annual citations and new authors in the field as impact indicators the paper concluded that after the NIH agreement the two categories which benefitted from lowering costs produced comparatively more citations and increased citations by new authors. The paper also concluded that lowering costs did not affect all stages from research to innovation equally and that greater openness of early-stage research leads to an increase in the diversity and the exploratory nature of follow-on innovation. The increased openness in sharing information about genetically engineered mice fosters innovation at various stages by encouraging researchers to explore new directions.

#### 3.5.3. Summary of the economic impact of open methods

Although only two papers were identified that studied open methods, both suggest benefits. One emphasized efficiency gains and innovation driven by time savings, the other stressed that lowering costs impact innovation because not only they enhance academic research but do so by speeding up access to innovation and support exploring new directions.

## 4. Discussion

The literature is abundant with theoretical considerations regarding the economic value of OS. Conceptually, this value is founded on the premise that the creation of new knowledge ultimately drives economic growth, while openness fosters a positive feedback loop: the more knowledge is openly accessible, the greater its potential to facilitate the generation of further knowledge. The scarce empirical evidence seems to substantiate the claim that OS positively impacts the economy. This is evidenced through case studies employing interviews and surveys and the comparatively higher prevalence of OS references in patent literature. In more detail:

— *The measured impacts found are all but one positive,* suggesting OS contributes to growth. The sole exception regarding OFD in the case of archaeology [39] where the authors calculated a negative net economic value when monetizing results.
— *Economic benefits are generated by efficiency gains, in terms of time and cost saved proxied by salaries and by contribution to innovation.* The latter is measured by patents and new products developments. *The most precise impacts have been identified in detailed studies by large repositories*, which offer free access to data and publications and have an interest in proving the value of their business model.
— *Macroeconomic benefits can be assessed through standard growth models* enriched by ‘accessibility and efficiency parameters’. The difficulty here obviously lies in determining the necessary parameters to run the simulations and the attribution claim.

Many of these studies rely on depositors’ and users’ surveys and on individual cases to empirically estimate impacts. This methodology however shows several limitations which the authors themselves recognize. First, respondents to these surveys are not a representative sample of the data services users, but a self-selected sub-group. One might reasonably expect that those taking the time to respond are likely to use and value data and services more than those that did not respond, which may suggest a risk of overestimation. Moreover, stated preferences need to be elicited following a strict and complex protocol, in order to be considered reliable and avoid a risk of hypothetical bias, especially in addressing the counterfactual problem that may require measuring the time to generate again the same data made openly available. When datasets or repositories are as peculiar as the ones analysed in the reported papers, it may be difficult if not impossible to identify a proper counterfactual (or different counterfactual scenarios may exist depending on the type and frequency of use) and therefore respondents may not be in a position to provide a reliable answer.

This indicative evidence is not ubiquitous and there are still important knowledge gaps: (i) the number of studies investigating economic impacts of OS is limited and concentrated in specific organizations, industries or countries; (ii) collecting and/or generating raw data is costly and only few organizations have been willing to invest in understanding the value of their openness strategy; (iii) the methods used are very dependent on surveys and interviews, hence subject to biases; (iv) some studies lack explicit methodological transparency, making it difficult to assess the validity and rigour of the implied estimations.

Analysing the frequency of various components within the literature reveals several noteworthy patterns:

— *In terms of disciplines*, biomedical research represents the most frequently studied domain, theoretically [16,69] and empirically as reported above. This can be attributed to a longstanding culture of data sharing, strong regulatory frameworks and global health relevance. Other sectors, which are reported in the literature, but with less empirical evidence, are agriculture [23], fisheries [15], AI tools and machine learning [70].
— *In terms of timing*, the literature which was found relevant for our purposes shows a slightly increasing trend. Since 2021 there are eight relevant papers, which constitute 30% of the total between 2006 and 2023.
— *In terms of numbers and prolificness of authors*, there are only two authors (Neil Beagrie and John Houghton) who have invested substantial effort in developing and applying methodologies both for OA and OFD; all the others have published one or two papers each in the specific topic. This reflects the highly relevant role the prolific authors play and the need for them to train their successors. Regardless of the reasons for the limited engagement of researchers in assessing the economic impact of OS, it is evident that further investigation is required to achieve a comprehensive understanding of the subject.
— *In terms of geographic distribution*, the majority of the evidence has been gathered from the USA and the UK. Many authors agree that international collaboration increases the likelihood of OA publication from the Global South and better integrating researchers from these countries into global innovative activities [58,71,72].

Our literature review points at important gaps and promising topics for research in OS. Prolific theorizing has helped set the stage but falls short of proving economic impact on its own. To truly demonstrate the economic value of OS, much more evidence is needed from models or case studies to capture the full extent of its effects. Expanding the tested methods (surveys, interviews and growth models) to unexplored areas—different sectors, countries and data repositories—could significantly enrich our understanding of the economic impact of OS. Similarly, other relevant economic factors, such as employment, are essential components for thoroughly understanding economic impacts and are currently missing completely.

An additional significant gap in research is how OS and private industry can mutually benefit. The question of how companies can contribute to OS, and how OS can support business needs, remains largely underexplored. Finding effective ways to balance the need for proprietary knowledge at the micro level with the widely recognized (albeit mostly theoretical) macroeconomic benefits of OS is an urgent research priority. Case studies by both large corporations and tech-oriented SMEs could provide initial insights before broader quantification efforts are pursued.

Finally, developing new methods and tools to assess economic impacts is essential—particularly for areas like citizen science and open evaluation, where there is barely any evidence at present. Developing systematic assessments could enable cost-benefit analyses for capturing its impacts in the future.

## 5. Conclusion

Based on a relatively low number of papers identified, the review confirms that the OS paradigm shift holds transformative potential for economic development through efficiency gains, innovation enhancement and economic growth. By enabling unrestricted access to research outputs, OS accelerates the pace of discovery and application, reducing redundancies and fostering collaboration across disciplines and sectors.

Efficiency gains are a hallmark of OS economic benefits. OFD eliminate barriers to data access, allowing researchers and industries to save time and financial resources. Case studies such as the Human Genome Project and other life science experiments demonstrate that open data repositories catalyse research, with downstream effects on innovation and economic growth. Similarly, OSS contributes to productivity by lowering costs and enabling businesses to optimize internal resources.

Innovation is another critical outcome of OS. Enhanced access to research outputs drives the development of new products and services, particularly in fields like biomedicine and IT. OSS, for example, fosters collaborative environments where startups and established firms can innovate more rapidly. Open methods, though less studied, reveal promising potential to accelerate scientific discoveries, as demonstrated in precompetitive collaborations in drug development.

Economic growth, while harder to quantify, is evident through macroeconomic analyses. Studies highlight significant returns on investment in OS initiatives, such as OA publications and data repositories.

Despite these encouraging findings, the empirical evidence base remains limited and unevenly distributed across sectors and geographies. Most studies focus on high-resource settings, leaving gaps in understanding OS’s impacts in developing economies where its benefits might be even greater. Additionally, methodologies often rely on self-reported data, introducing biases that challenge the reliability of results. Areas such as citizen science and open evaluation remain underexplored, emphasizing the need for new tools and metrics to assess their impacts.

Moving forward, researchers and policymakers should encourage the development of standardized metrics to measure the economic impacts of OS. Expanding research to include diverse sectors and regions will enhance our understanding of OS’s global potential.

## Data Availability

Datasets and additional materials supporting this article are published on Zenodo [73]. Supplementary material is available online [74].

## Footnotes

^1^
Budapest Open Access Initiative Declaration in 2002 and the Berlin Declaration on Open Access to Knowledge in the Sciences and Humanities in 2003.

^2^
The US National Institutes of Health (NIH) was one of the first major funding agencies to mandate open access to research. Many more followed including the European Commission.

^3^
The public nature of knowledge is given by its inherent properties of being (i) non-rivalry: knowledge can be shared indefinitely without being depleted since a person’s use of knowledge does not reduce its availability for others and (ii) non-excludable—once knowledge is made available, it is difficult (or costly) to prevent others from accessing or using it.

^4^
The corresponding search terms in the automatic process were used with an asterisk as presented in table 1 to capture all derivatives of the respective words.

^5^
‘Multifactor productivity (MFP) reflects the overall efficiency with which labour and capital inputs are used together in the production process. Changes in MFP reflect the effects of changes in management practices, brand names, organisational change, general knowledge, network effects, spillovers from production factors, adjustment costs, economies of scale, the effects of imperfect competition and measurement errors. Growth in MFP is measured as a residual, i.e. that part of GDP growth that cannot be explained by changes in labour and capital inputs. In simple terms therefore, if labour and capital inputs remained unchanged between two periods, any changes in output would reflect changes in MFP. This indicator is measured as an index and in annual growth rates’ [34].

^6^
The Human Genome Project was a landmark international scientific research initiative aimed at mapping and understanding all the genes in the human genome (the ‘genome'). The project began in 1990 and was completed in 2004, ahead of its original schedule. It stands as one of the most significant achievements in modern biology and genomics. The sequencing made simultaneous use of two different approaches with the competition and collaboration between HGP, a publicly funded international collaboration, and Celera Genomics, a private company. Celera and the HGP competed to finish the genome first. However, they eventually collaborated by sharing data, with Celera using HGP’s publicly available mapped sequences to improve its assembly process.

^7^
For example, Wadman [50] has addressed these concerns.

^8^
There are two main routes to making research outputs openly accessible. One involves publishing articles or books via the OA route on a publisher’s platform (often referred to as gold open access). The other involves archiving a version of the manuscript in an OA repository (often described as green open access). Content published via the gold OA route is accessible immediately on publication, while manuscripts deposited via the green OA route may, in many cases, be made accessible only once a self-archiving embargo period has elapsed. The terms for onward sharing and re-use of OA content will depend on the license under which it has been made available [59].

^9^
The Solow–Swan model is a highly respected economic model that explains long-term economic growth by examining capital accumulation, labour or population growth, and technological progress. Developed independently by Robert Solow and Trevor Swan in the 1950s, the model primarily focuses on how these factors contribute to a nation’s output or GDP over time.

^10^
Gerd is gross expenditure on research and development (all sectors); GovERD is government expenditure on research and development (a sub-total of GERD).

^11^
GitHub (https://github.com) is a developer platform to build, scale and deliver secure software.

## Appendix A. Overview of indicators on the efficiency of large public open repositories

Table 3.

**Table 3.** The table compares standard indicators on costs and benefits of large public open repositories.

indicators	ADS (Archaeology Data Service)	BADC (British Atmospheric Data Centre)	ESDS (Economic and Social Data Service)	EMBL-EBI (European Bioinformatics Institute of the European Molecular Biology Laboratory)	Protein Data Bank	HGP
annual investment value	£1.2 million	£2.8 million	£23 million	£110 million	$6.9 million	
annual use value	£1.4 million	£2.3 million	£24 million	£5.5 billion	$5.5 billion	
cost of obtaining	£1 439 091	£2 257 170	£4 million			
cost of provision	£698 000	£2 049 600	£3 million			
annual users’ willingness to pay	£1.1 million	£5.2 million	£25 million	£1.25 billion	$760 million	
annual willingness to accept	£7.4 million	£15 million	£81–111 million	£68 600 per respondent	—	
consumer surplus	−£312 000	£3 million	£21 million	—	—	1988–2010 $796 billion
net economic value	−£1 million	£935 000	£18 million	—	—	
user efficiency gain (minimum per year)	£13 million	£10 million	£68 million	£2.6 billion	$2 billion	
user efficiency gain (maximum per year)	£58 million	£58 million	£112 million	£11 billion	$3 billion	
return on investment (additional use, NPV over 30 years)	£2.4 million to £9.7 million	£11 million to £34 million	£58 million to £233 million	£9 billion	$8 billion	
return on investment (additional use)	2.1- to 8.3-fold	4- to 12-fold	2.5- to 10-fold	—	—	
return on investment (non-recreate, cannot be easily recreated by others)	£1.5 million to £5.9 million (1.3 to 5.1-fold RoI)	—	—	£6 billion per annum from reduced duplication of effort.	—	

## References

[1] Klebel T, Traag V, Grypari I, Stoy L, Ross-Hellauer T. 2024 The academic impact of open science: a scoping review. SocArXiv (10.31235/osf.io/ptjub) PMC11879623 40046663

[2] Cole NL, Kormann E, Klebel T, Apartis S, Ross-Hellauer T. 2024 The societal impact of open science: a scoping review. R. Soc. Open Sci. **11**, 240286. (10.1098/rsos.240286) 39100167 PMC11296153

[3] OECD. 2015 Making open science a reality. Technology and Industry Policy Papers no. 25. OECD Publishing. (10.1787/5jrs2f963zs1-en)

[4] UNESCO. 2021 UNESCO recommendation on open science. See https://unesdoc.unesco.org/ark:/48223/pf0000379949.

[5] Romer PM. 1990 Endogenous technological change. J. Polit. Econ. **98**, S71–S102. (10.1086/261725)

[6] Arrow KJ. 1962 Economic welfare and the allocation of resources for invention. In The rate and direction of inventive activity: economic and social factors, pp. 609–626. Princeton, NJ: Princeton University Press. (10.1515/9781400879762-024)

[7] Stiglitz JE. 1999 Knowledge as a global public good. In Global public goods: international cooperation in the 21st century (eds K Inge, G Isabelle, S Marc), pp. 308–325. Oxford, UK: Oxford University Press. (10.1093/0195130529.003.0015)

[8] David P. 1998 Common agency contracting and the emergence of ‘open science’ institutions. Am. Econ. Rev. **88**, 15–21. http://www.jstor.org/stable/116885

[9] David P. 2003 The economic logic of ‘open science’ and the balance between private property rights and the publicdomain in scientific data and information: a primer. Discussion Paper No. 02-30. SIEPR.

[10] David P. 2004 Understanding the emergence of ‘open science’ institutions: functionalist economics in historical context. Ind. Corp. Chang. **13**, 571–589. (10.1093/icc/dth023)

[11] Suber P. 2003 The taxpayer argument for open access. Washington, DC: SPARC Open Access Newsletter.

[12] Chataway J, Parks S, Smith E. 2017 How will open science impact on university-industry collaborations? Foresight STI Gov. **11**, 44–53. (10.17323/2500-2597.2017.2.44.53)

[13] Yozwiak NL, Schaffner SF, Sabeti PC. 2015 Data sharing: make outbreak research open access. Nature **518**, 477–479. (10.1038/518477a) 25719649

[14] Mazzucato M. 2011 The entrepreneurial state. Soundings **49**, 131–142. (10.3898/136266211798411183)

[15] McManamay RA, Utz RM. 2014 Open‐Access databases as unprecedented resources and drivers of cultural change in fisheries science. Fisheries **39**, 417–425. (10.1080/03632415.2014.946128)

[16] Harding RJ. 2017 Open science and accelerating discovery in rare and neglected diseases. In Expanding perspectives on open science: communities, cultures and diversity in concepts and practices, pp. 1–5. Amsterdam, The Netherlands: IOS Press. (10.3233/978-1-61499-769-6-1)

[17] Chan GR. 2015 Cost impact in managing the transition to an open access model. See https://docs.lib.purdue.edu/cgi/viewcontent.cgi?article=1558&context=charleston.

[18] Williams RJ, Walker I, Takle AK. 2012 Collaborative approaches to anticancer drug discovery and development: a Cancer Research UK perspective. Drug Discov. Today **17**, 185–187. (10.1016/j.drudis.2012.01.020) 22314099

[19] van Vlijmen H *et al*. 2020 The need of industry to Go FAIR. Data Intell. **2**, 276–284. (10.1162/dint_a_00050)

[20] Mueller-Langer F, Andreoli-Versbach P. 2018 Open access to research data: strategic delay and the ambiguous welfare effects of mandatory data disclosure. Inf. Econ. Policy **42**, 20–34. (10.2139/ssrn.2458362)

[21] Tennant JP, Waldner F, Jacques DC, Masuzzo P, Collister LB, Hartgerink ChrisHJ. 2016 The academic, economic and societal impacts of Open Access: an evidence-based review. F1000Research **5**, 632. (10.12688/f1000research.8460.1) 27158456 PMC4837983

[22] Arzberger P, Schroeder P, Beaulieu A, Bowker G, Casey K, Laaksonen L, Moorman D, Uhlir P, Wouters P. 2004 Promoting access to public research data for scientific. Econ. Soc. Dev. Data Sci. J. **3**, 135–152. (10.2481/dsj.3.135) 15031482

[23] Ali B, Dahlhaus P. 2022 The role of FAIR data towards sustainable agricultural performance: a systematic literature review. Agriculture **12**, 309. (10.3390/agriculture12020309)

[24] Wehn U *et al*. 2021 Impact assessment of citizen science: state of the art and guiding principles for a consolidated approach. Sustain. Sci. **16**, 1683–1699. (10.1007/s11625-021-00959-2)

[25] Fell MJ. 2019 The economic impacts of open science: a rapid evidence assessment. Publications **7**, 46. (10.3390/publications7030046)

[26] Ali-Khan SE, Jean A, MacDonald E, Gold ER. 2018 Defining success in open science. MNI Open Res. **2**, 2. (10.12688/mniopenres.12780.2) PMC5852639 29553146

[27] Ross-Hellauer T, Klebel T, Pitelis A. 2022 Protocol for scoping reviews of (1) academic, (2) societal and (3) economic impacts of open science. Open Science Framework. (10.17605/OSF.IO/GM57C)

[28] Cole NL, Klebel T, Ross-Hellauer T. 2023 Addendum to protocol for scoping reviews of (1) academic, (2) societal and (3) economic impacts of open science: protocol for grey literature search and indexing of sources, and for screening and data charting in the syrf platform. Open Science Framework. See https://osf.io/3b6xj.

[29] Ikkatai Y, Ono E. 2018 Exploring characteristics of academic crowdfunding in Japan. In 2018 7th Int. Congress on Advanced Applied Informatics (IIAI-AAI), Yonago, Japan. (10.1109/iiai-aai.2018.00098)

[30] Chesbrough H. 2003 Open innovation: the new imperative for creating and profiting from technology. Boston, MA: Harvard Business School Press.

[31] Bikard M, Vakili K, Teodoridis F. 2019 When collaboration bridges institutions: the impact of university–industry collaboration on academic productivity. Organ. Sci. **30**, 426–445. (10.1287/orsc.2018.1235)

[32] Arnone S, Buoni L, Manieri A, Mathieu PP, Mazzitelli G, Spagnoli F. 2016 University, industry, and research cooperation: the Lazio Pulse Initiative. In Presented at the International Technology, Education and Development Conference, pp. 3808–3814. Seville, Spain. (10.21125/iceri.2016.1902)

[33] Eurostat O. 2018 Oslo manual 2018: guidelines for collecting, reporting and using data on innovation, 4th edn. OECD. See https://www.oecd.org/en/publications/oslo-manual-2018_9789264304604-en.html.

[34] OECD. 2015 Multifactor productivity. See https://www.oecd.org/en/data/indicators/multifactor-productivity.html?oecdcontrol-00b22b2429-var3=2021.

[35] Solow R. 1957 Technical change and the aggregate production function. Rev. Econ. Stat. **39**, 312–320.

[36] Abramovitz M. 1956 Resource and output trends in the United States since 1870. Am. Econom. Rev **46**, 5–23.

[37] Jorgenson D, Griliches Z. 1967 The explanation of productivity change. Rev. Econ. Stud **34**, 249–283.

[38] Beagrie N, Houghton J. 2012 Economic impact evaluation of the economic and social data service. Economic and Social Research Council.

[39] Beagrie N, Houghton J. 2013 The value and impact of the Archaeology Data Service: a study and methods for enhancing sustainability. See https://repository.jisc.ac.uk/5509/1/ADSReport_final.pdf.

[40] Beagrie N, Houghton J. 2013 The Value and Impact of the British Atmospheric Data Centre. See https://vuir.vu.edu.au/39092/1/2013_Beagrie%26Houghton_Value%26Impact_Brit_Atmospheric_Data_Centre.pdf.

[41] Beagrie N, Houghton J. 2014 The value and impact of data sharing and curation: a synthesis of three recent studies of UK research data centres. JISC.

[42] Beagrie N, Houghton J. 2016 The Value and Impact of the European Bioinformatics Institute. See https://www.embl.org/documents/wp-content/uploads/2021/09/EMBL-EBI_Impact_report-2016-summary.pdf.

[43] Beagrie N, Houghton J. 2021 Data-driven discovery: the value and impact of EMBL-EBI managed data resources. EMBL-EBI.

[44] Sullivan KP, Brennan-Tonetta P, Marxen LJ. 2017 Economic impacts of the research collaboratory for structural bioinformatics (RCSB) protein data bank. RCSB Protein Data Bank 2017. (10.2210/rcsb_pdb/pdb-econ-imp-2017)

[45] Williams HL. 2013 Intellectual property rights and innovation: evidence from the human genome. J. Polit. Econ. **121**, 1–27. (10.1086/669706) PMC3955392 24639594

[46] Arshad Z *et al*. 2016 Open access could transform drug discovery: a case study of JQ1. Expert Opin. Drug Discov. **11**, 321–332. (10.1517/17460441.2016.1144587) 26791045

[47] Giovani B. 2017 Open data for research and strategic monitoring in the pharmaceutical and biotech industry. Data Sci. J **16**, 1–9. (10.5334/dsj-2017-018)

[48] Nielsen AF, Michelmann J, Akac A, Palts K, Zilles A, Anagnostopoulou A, Langeland O. 2023 Using the future wheel methodology to assess the impact of open science in the transport sector. Sci. Rep **13**, 1–15. (10.1038/s41598-023-33102-5) 37046066 PMC10097618

[49] Tripp S, Grueber M. 2011 The economic impacts of human genome project. TEConomy Partners, LLC.

[50] Wadman M. Economic return from Human Genome Project grows. Nature New Biol. (10.1038/nature.2013.13187)

[51] Houghton J, Swan A, Brown S. 2011 Access to research and technical information in Denmark. See https://eprints.soton.ac.uk/272603/.

[52] Coccia M. 2021 Evolution and structure of research fields driven by crises and environmental threats: the COVID-19 research. Scientometrics **126**, 9405–9429. (10.1007/s11192-021-04172-x) 34720251 PMC8541882

[53] Look H, Marsh K. 2012 Benefits of open access to scholarly research outputs to the public sector. In A report for the open access implementation group. See https://wiki.lib.sun.ac.za/images/e/e3/Report-to-oauk-benefits-of-open-access-public-sector.pdf.

[54] Houghton J, Rasmussen B, Sheehan P, Oppenheim C, Morris A, Creaser C, Greenwood H, Summers M, Gourlay A. 2009 Economic implications of alternative scholarly publishing models: exploring the costs and benefits. See https://vuir.vu.edu.au/15222/1/EI-ASPM_Report.pdf.

[55] Bryan KA, Ozcan Y. 2021 The impact of open access mandates on invention. Rev. Econ. Stat. **103**, 954–967. (10.1162/rest_a_00926)

[56] Jahn N, Klebel T, Pride D, Knoth P, Ross-Hellauer T. 2022 Quantifying the influence of open access on innovation and patents. Open Res. Eur. **2**, 64. (10.12688/openreseurope.14680.1)

[57] Houghton J, Sheehan P. 2006 The economic impact of enhanced access to research findings. Victoria University.

[58] Houghton J, Sheehan P. 2009 Estimating the potential impacts of open access to research findings. Econ. Anal. Policy **39**, 127–142. (10.1016/s0313-5926(09)50048-3)

[59] Nature S. 2025 Green or Gold routes to open access. See https://www.springernature.com/gp/open-science/about/green-or-gold-routes-to-oa.

[60] Nagle F. 2019 Open source software and firm productivity. Manag. Sci. **65**, 1191–1215. (10.1287/mnsc.2017.2977)

[61] Conti A, Peukert C, Roche M. 2021 Beefing IT up for your Investor? Engagement with open source communities, innovation and startup funding: evidence from GitHub. SSRN Electron. J. (10.2139/ssrn.3883936)

[62] Chesbrough H. 2023 Measuring the economic value of open source: a survey and a preliminary analysis. The Linux Foundation.

[63] European Commission: Directorate-General for Communications Networks, Content and Technology. 2021 The impact of open source software and hardware on technological independence, competitiveness and innovation in the EU economy. European Commission. See https://data.europa.eu/doi/10.2759/430161.

[64] Laurent A. 2004 Understanding open source and free software licensing. Sebastopol, CA: O’Reilly Media.

[65] Calderón JBS, Robbins CA, Korkmaz G, Kramer BL. 2022 Measuring the cost of open source software innovation on GitHub. Bureau of Economic Analysis.

[66] Korkmaz G. 2020 *Measuring the cost and impact of open source software innovation on GitHub*. Presentation at Harvard Business School.

[67] Lee WH. 2015 Open access target validation is a more efficient way to accelerate drug discovery. PLoS Biol. **13**, e1002164. (10.1371/journal.pbio.1002164) 26042736 PMC4456377

[68] Murray F, Aghion P, Dewatripont M, Kolev J, Stern S. 2016 Of mice and academics: examining the effect of openness on innovation. Am. Econ. J. **8**, 212–252. (10.1257/pol.20140062)

[69] Arefolov A *et al*. 2021 Implementation of the FAIR data principles for exploratory biomarker data from clinical trials. Data Intell. **3**, 631–662. (10.1162/dint_a_00106)

[70] Chen SC, Chen YC, Chen WL. 2017 Data mining techniques vs. policy development: evaluating advanced applied technological policies and emerging communication technology. In 2017 IEEE International Symposium on Multimedia (ISM). Presented at the 2017 IEEE International Symposium on Multimedia (ISM), pp. 469–474. Taichung: IEEE. (10.1109/ism.2017.93)

[71] Iyandemye J, Thomas MP. 2019 Low income countries have the highest percentages of open access publication: a systematic computational analysis of the biomedical literature. PLoS One **14**, e0220229. (10.1371/journal.pone.0220229) 31356618 PMC6663019

[72] Boufarss M, Laakso M. 2023 Open access and international coauthorship: a longitudinal study of the United Arab Emirates research output. Quant. Sci. Stud. **4**, 372–393. (10.1162/qss_a_00256)

[73] Liarti S, Tsipouri L, Vignetti S, Martins Grapengiesser I. 2025 Data from ‘The economic impact of open science: a scoping review’ (Version 1) [Data set]. Zenodo. (10.5281/zenodo.14892767) PMC12441599 40969686

[74] Tsipouri L, Liarti S, Vignetti S, Grapengiesser IM. 2025 Supplementary material from: The Economic Impact of Open Science: A Scoping Review. Figshare. (10.6084/m9.figshare.c.8003559) PMC12441599 40969686

